# Association of GHR Polymorphisms with Milk Production in Buffaloes

**DOI:** 10.3390/ani10071203

**Published:** 2020-07-15

**Authors:** Shymaa M. El-Komy, Ayman A. Saleh, Tamer M. Abdel-Hamid, Mohammed A. El-Magd

**Affiliations:** 1 Department of Animal Production, Faculty of Agriculture, Tanta University, Tanta 31527, Egypt; dr_shymaelkomy@outlook.com; 2 Department of Animal Wealth Development, Veterinary Genetics & Genetic Engineering, Faculty of Veterinary Medicine, Zagazig University, Zagazig 44519, Egypt; aymangene@zu.edu.eg; 3 Department of Animal Wealth Development, Animal Breeding and Production, Faculty of Veterinary Medicine, Zagazig University, Zagazig 44519, Egypt; tamer@zu.edu.eg; 4 Department of Anatomy & Embryology, Faculty of Veterinary Medicine, Kafrelsheikh University, Kafrelsheikh 33516, Egypt

**Keywords:** growth hormone receptor, milk yield, milk quality, single nucleotide polymorphisms

## Abstract

**Simple Summary:**

The present study reported two missense mutations in the buffalo *GHR* gene: A novel (c.380G>A) and (c.836T>A) which was described in previous studies. These two single nucleotide polymorphisms (SNPs) were found to be associated with milk yield, fat %, protein %, and 305 day-milk, fat and protein yield, with higher performance for AA haplotype animals. Therefore, selection of buffaloes with AA haplotype would more likely improve milk production traits. Consequently, this would allow breeders to take more precise selection decisions, leading to significantly higher productivity and profitability within the Egyptian buffalo herds.

**Abstract:**

For its role in the mediation of growth hormone (GH) galactopoietic effect, growth hormone receptor (*GHR*) was considered a functional candidate gene for milk performance in cattle. However, its genetic variation and potential effect have not been investigated in Egyptian buffaloes. This study aimed to screen *GHR* for polymorphisms and study their associations with milk traits in Egyptian buffaloes. Polymerase chain reaction, single-strand conformation polymorphism, and sequencing were used to identify mutations in 4 exons (E4–E6 and E8) of the *GHR* gene in 400 Egyptian buffaloes. No polymorphisms were found in E4, while 2 SNPs (c.380G>A/p.Arg127Lys and c.387C>T/p.Gly129) in E5, one silent mutation (c.435A>G/p.Pro145) in E6, and another missense mutation (c.836T>A/p.Phe279Tyr) in E8 were detected. The c.380G>A SNP in the extracellular domain was associated with milk yield, fat %, protein %, and 305-day milk, fat and protein yield, with higher levels in animals carrying the mutant A allele. The c.836T>A SNP in the transmembrane domain was associated with milk yield, fat %, protein %, and 305-day milk, fat and protein yield, with higher milk yield and lower fat %, protein %, fat and protein yield in the mutant A allele-animals. Interestingly, animals with the two mutant AA alleles produced higher milk yield, fat %, protein %, fat and protein yield, accompanied with upregulated expressions of GHR, GH, insulin-like growth factor 1 (IGF1), prolactin (PRL), prolactin receptor (PRLR), β-casein (encoded by CSN2 gene), and diacylglycerol acyltransferase-1 (DGAT1) genes and proteins in milk somatic cells. Therefore, selection of Egyptian buffaloes with mutant AA haplotypes for the novel c.380G>A SNP and the well-known c.836T>A SNP could improve milk yield and quality in buffaloes.

## 1. Introduction

Low milk production of buffaloes is one of the most challenging problems on Egyptian dairy farms, which is mainly attributable to poor environmental conditions, malnutrition, and poor genetic capabilities. Milk production and composition are complex traits that are coregulated by both: Environmental factors (e.g., herd, season, stage of lactation, and diet) and genes [1]. The growth hormone receptor (*GHR*) gene is one of the most important genes that mediates most, or even all, of the growth hormone (GH) effect. GHR protein is a member of the type 1 cytokine/hematopoietin receptor family, that consists of signal sequence, extracellular (harboring the GH-binding site), transmembrane, and long intracellular (participating in GH signaling) domains [2]. Cattle *GHR* gene is mapped to chromosome 20 and comprises 10 exons (E), of which E1 is very small and has non-coding sequences [3,4]. 

Many studies screened *GHR* for potential polymorphisms and reported their effects on milk production and quality [5,6,7]. The genome-wide association study (GWAS) identified milk performance-related quantitative trait loci (QTL) and suggested *GHR* as a strong functional and structural candidate gene for this QT [5,7]. A non-synonymous SNP in *GHR* E8 (c.836T>A, p.Phe279Tyr) is responsible for the substitution of phenylalanine (neutral aa) with tyrosine (polar un-charged aa), in the transmembrane domain of the GHR protein. This substitution was significantly associated with milk production and milk fat and protein contents [5,6,8,9]. Animals with the mutant A allele produce higher milk yield, but lower fat and protein yields than those with the T allele [5,6,7,10]. Although most of these previous studies identified c.836T>A as a causative SNP for milk production QTL in cattle, some other SNPs such as a silent mutation (SNP c.463C>T, p.Leu155) in E6 of bovine *GHR* were also significantly associated with higher milk yield and superior milk quality (high protein, casein, and fat yields and percentages) with higher milk coagulation properties and lower somatic cells score [11].

Although several studies have detected QTL and candidate genes related to milk production and composition in cattle, to date, there are nearly no available data on QTLs linked to milk production and composition traits in water buffalo. In their recent genome-wide search for mutations associated with Murrah buffalo economic traits, Surya et al. [12] found 483 SNPs in 66 genes affecting milk traits. Among these SNPs, 35 SNPs were found in the *GHR* locus (NW_005785241.1 from nt 844295 to1151290): 9 in the promotor, 4 in intron2, 13 in intron3, 2 in intron4, 3 in E5, and 4 in inton6). All of these SNPs led to silent mutations except c.381A>C SNP in E5 which resulted in arginine to serine substitution (p.Arg127Ser). In E5, other silent mutations were c.348T>C and c.387C>T. Unfortunately, this previous study did not investigate the effect of the causative mutation c.836T>A in Murrah buffalo. However, another earlier study by Shi et al. [13] confirmed the presence of this SNP in Indian water buffaloes and Chinese swamp buffaloes; however, only two genotypes (TT and AT) were detected in these 136 buffaloes. Again, Shi et al. [13] did not study the effect of this important SNP on milk production and milk quality traits in buffaloes. Although many studies reported the ability of noncoding polymorphisms including: synonymous, intergenic and intronic SNPs to modify complex traits in animals [14,15,16], it is still essential to study non-synonymous SNPs, as they could directly alter the amino acids sequence, which could possibly lead to phenotypic variation. Therefore, in this study, we screened coding sequences of E4-E6 and E8 for polymorphisms. These exons were selected due to their polymorphic nature as revealed in previous publications in cattle and buffalo [5,9,12,13].

As mentioned above, some *GHR* SNPs were found in Indian and Chinese buffaloes, but their associations with milk performance have not been studied yet. Moreover, no information has been available for *GHR* genetic variations and their effects on milk performance in Egyptian water buffaloes. Additionally, milk traits association studies on cattle *GHR* did not explore the functional effect of these SNPs on expression of genes and proteins related to milk performance. Hence, in the present study, we screened Egyptian buffaloes *GHR* for genetic variations and analyzed their associations with milk yield, milk quality, and molecular changes.

## 2. Materials and Methods

This study was conducted in the molecular biology lab (MBL), Faculty of Veterinary Medicine, Kafrelsheikh University (funded by STDF, Egypt). The study protocol was reviewed and approved by Kafrelsheikh University Animal Care and Ethics Committee with an ethical approval number of KFS 127/9.

### 2.1. Animals and Samples Preparation

Four hundred pure dairy Egyptian water buffaloes were randomly selected from El-Nataff El-Gidid Experimental Stations (Mahalet Mousa, Kafrelsheikh Governorate). All animals were managed under similar feeding and housing conditions. These pure Egyptian buffaloes were daughters of 80 sires, with 2 to 30 daughters per sire. These animals were milked twice daily (with 12 h interval) and fed concentrate mixtures. All phenotypic data of lactation traits (total milk yield per 305-day lactation (ranged from 1707–2239 kg) and lactation length (260–360 days)) were obtained from farm official records covering the period from February 2014 to December 2018. A total of 10,130 milk samples from 1224 lactations (lactations 1 to 5 and parities 1 to 5) of all selected buffaloes were collected during monthly test-day milk recording. Milk composition including fat %, protein %, lactose %, and total solid % were analyzed in 400 samples (one sample per buffalo) using Automated Funke Gerber Lactoflash Dairy Analyser (Berlin, Germany) in the Molecular Biology Laboratory (MBL). The 305-day milk, fat and protein yield were calculated based on data of 305-day milk, fat %, and protein % for animals.

### 2.2. Sample Preparation

Blood samples (5 mL/animal) were collected from the jugular veins of all animals (n = 400) into EDTA-coated tubes (for DNA extraction). Milk samples for real-time PCR and western blot (n = 9/haplotype) were collected from animals at the same lactation age (5 years), and stage (early stage at the 57th–63rd day of lactation). Milk somatic cells pellets (for RNA and protein extraction) were isolated from milk samples (1 L/animal) by double centrifugations (the first at 1500× *g* for 30 min to get rid of fat and the second at 1100× *g* for 15 min) with an interval of phosphate buffer saline wash.

### 2.3. SNPs Detection and Genotyping

SNPs were detected in genomic DNA samples using polymerase chain reaction (PCR)-single strand conformation polymorphism (SSCP) and sequencing. DNA extraction was performed from 200 µL whole blood sample using a commercial kit (GeneJET genomic DNA extraction kit, Thermo Scientific, #K0721, Waltham, MA, USA), following the manufacturer’s protocol. Four loci of the *GHR* gene containing E4-E6 and E8 were amplified by PCR using primers and annealing temperatures as presented in Table 1. PCR mix and thermal cycle conditions were done as previously described [17] but with an annealing temperature of 56 °C for 40 s. The specificity and quality of PCR bands were validated using 1% agarose gels.

SNPs genotyping was done by SSCP which was carried out as previously described [18,19]. Animals were genotyped based on the pattern of SSCP bands. A total of 77 purified PCR products (7 from each SSCP banding pattern) were sequenced in both directions by outsourcing (Macrogen Company, Seoul, South Korea). Sequences analysis and amino acid alignment were done by Geneious 4.8.4 software (Biomatters, Ltd, Auckland, New Zealand).

### 2.4. Real-Time PCR

Milk somatic cells (SCs, mainly mammary epithelial cells in addition to few leukocytes) were successfully used to assess the expression of genes and proteins associated with milk yield and composition in goats [20,21]. SCs can be easily isolated from milk samples, unlike mammary gland epithelial cells. RNA was extracted from SCs samples (n = 9 /haplotype) by a commercial kit (GeneJET RNA Purification Kit, Thermo Scientific, # K0731, USA), according to the manufacturer’s instructions. The integrity and purity/concentration were assessed by 1% agarose gels and Nanodrop (Q5000, Quawell, San Jose, CA, USA), respectively. The RNA was reverse transcribed by RevertAid H Minus Reverse Transcriptase (Thermo Scientific, #EP0451, USA). The qPCR mix contained 2X Maxima SYBR Green Master Mix (Thermo Scientific, # K0221, USA), cDNA, and specific primers for *GHR*, *GH*, *IGF1*, *PRL*, *PRLR*, *CSN2*, and *DGAT1* genes (Table 1). These primers were constructed by primer 5.0 software based on GenBank available water buffalo sequences. Each sample was run in triplicate along with an internal control (*β-actin*) using StepOnePlus real-time PCR system (Applied Biosystem, Foster City, CA, USA). The conditions (time and temperature) of thermal cycles and melting curves were performed as previously described [22,23]. The gene expression (in form of fold change) was determined by the 2^−∆∆Ct^ method. Real-time PCR data obtained from SCs were validated by detection of *GHR*, *GH*, *IGF1*, *PRL*, *PRLR*, *CSN2*, and *DGAT1* in mammary gland tissues from emergency slaughtered animals (healthy animals suffered an accident that prevented its transport to the slaughterhouse, n = 3/haplotype, data not shown), which showed similar expression profile as SCs. No significant difference in expression of these genes was noticed between SCs and mammary tissues.

### 2.5. Western Blot

Western blot was used to determine the expression of GHR, GH, IGF1, PRL, PRLR, β-casein, and DGAT1 proteins and was done as previously described [24,25]. Briefly, somatic cells samples (in form of pellets) were lysed by RIPA lysis buffer, loaded into 10% SDS-PAGE gels, and proteins were transported to a 0.45 μm polyvinylidene fluoride membrane, which was incubated with the appropriate primary antibodies followed by the secondary antibody (see Table S1 for more details). Protein bands were normalized by β-actin protein and were quantified by Image J software (National Institutes of Health, Bethesda, MD, USA).

### 2.6. Statistical Analysis

Gene heterozygosity (He), effective allele numbers (Ne), and Hardy–Weinberg equilibrium (HWE), were calculated by PopGene 32, version 1.32 (Alberta, Canada). Polymorphism information content (PIC) was calculated with GenCal software. Haplotypes and haplotype frequencies were determined by PHASE 2.1 (Chicago, IL, USA). Linkage disequilibrium (LD) measured by LD coefficient (D’) and absolute association (r^2^) between the two SNPs, minor allele frequency (MAF), and haplotype reconstruction were estimated by Haploview 4.2 (Cambridge, MA, USA).

Associations of *GHR* SNPs and haplotypes with milk traits were determined using the following mixed linear model by SAS V9 (SAS Inst. Inc., Cary, NC, USA): y*_ijklmn_*= μ + Sire*_i_* + A*_j_* + P*_k_* + L*_l_* + M*_m_* + H*_n_* + e*_ijklmn_* where; y represents the value of milk yield and composition traits; μ is the overall mean for each trait; Sire*_i_* represents the random effects of the *i*th sires. A*_j_* is the fixed effect of the age of the *j*th animal at calving expressed in years (6 levels: 1 = <4 years, 2 = 4 years, 3 = 5 years, 4 = 6 years, 5 = 7 years, 6 = >7 years); P*_k_* is the fixed effect of the parity (three levels: parity 1, 2 and 3–5); L*_l_* is the fixed effect of the *l*th stage of lactation (10 levels of 30 days each); M*_m_* is the fixed effect of the *m*th month of calving (12 levels); H*_n_* is the fixed effect of the *n*th *GHR* haplotype with 4 levels (n = GT, GA, AT, and AA); and e*_ijklmn_* is the random residual effect.

Univariate analyses were used to test the association of the fixed effects and the dependent variables of interest setting a liberal *p*-value of (*p* < 0.25). Then, final significance for testing the fixed effects in the multivariable model was established at *p* < 0.05. The results of the multiple comparisons were corrected using Bonferroni correction, and the differences were considered significant at *p* < 0.05. Data were expressed as least squares means ± standard error of mean (SEM). Difference in expression levels of candidate genes and proteins among different haplotypes were plotted using GraphPad Prism 8 (GraphPad Software, Inc., San Diego, CA, USA).

## 3. Results and Discussion

The milk yield of Egyptian buffaloes is inferior when compared to foreign buffaloes due to many environmental, nutritional, and genetic causes. Lack of precise practices of marker-assisted selection (MAS) for valuable traits in Egypt is one of the most important reasons associated with inferior milk traits. Therefore, genetic improvement using MAS is necessary to improve the production traits. This study is part of a broader project targeting the genetic improvement of the of Egyptian water buffaloes. We previously screened *IGF1*, *IGF1R*, *IGF2*, *IGF2R*, and *Cyp19A1* for polymorphisms and analyzed their associations with growth traits and fertility [15,16,18,24,26]. Herein, we extended our investigation to identify genetic variations in the *GHR* gene and determine their potential effects on milk production traits.

### 3.1. Analysis of the Detected SNPs

PCR products from *GHR.E4* locus (Figure S1) were genotyped by SSCP and the obtained results revealed only one banding pattern (Figure S2), indicating a lack of genetic variations in this locus. This was additionally confirmed by sequencing (Figure S3). A lack of polymorphisms in buffalo *GHR.E4* was also reported in other studies [4,12].

**Figure 1:**
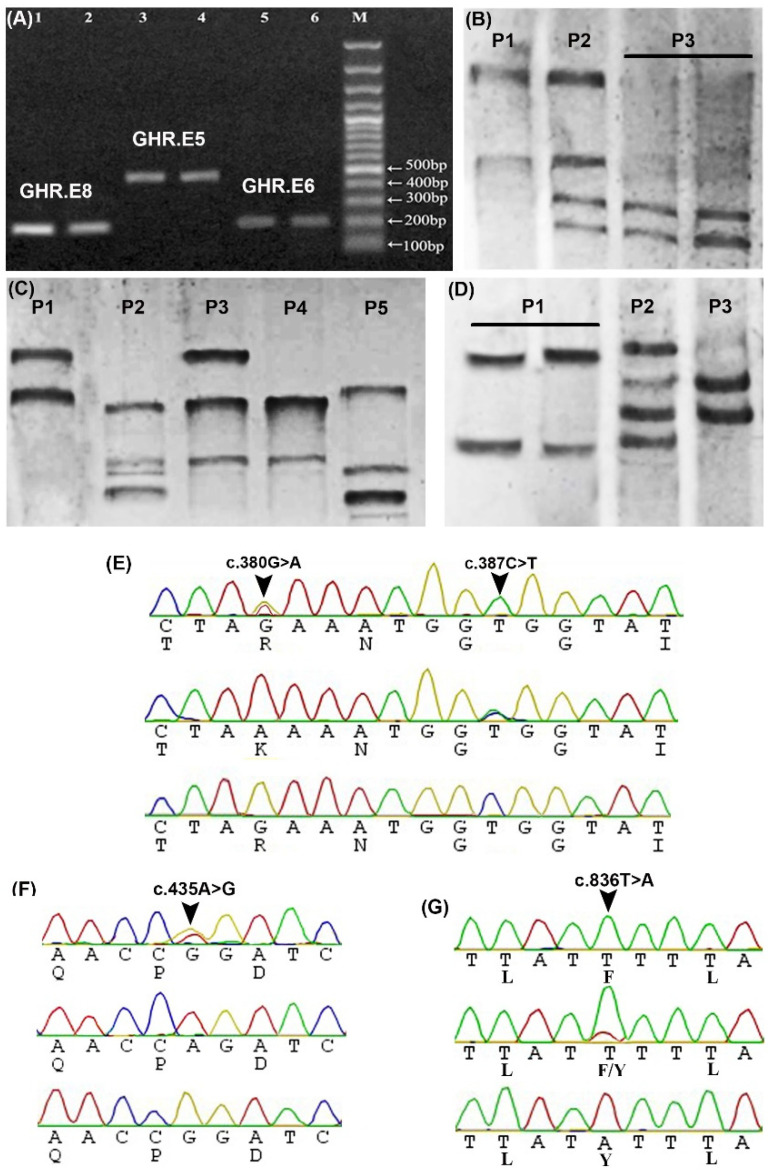

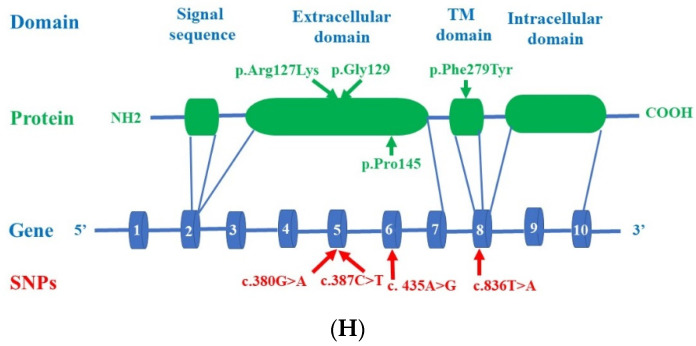
Detection of *GHR* SNPs using PCR- single strand conformation polymorphism (SSCP) and sequencing. (**A**) Agarose gel shows amplified fragments of *GHR.E6* (205 bp, lanes 5 and 6), *GHR.E5* (472 bp, lanes 3 and 4), and *GHR.E8* (166 bp, lanes 1 and 2) from two different samples, M: 100 bp DNA ladder. (**B**) PCR-SSCP bands of *GHR.E6* show three different patterns (P1-P3) in four different samples. (**C**) PCR-SSCP bands of *GHR.E5* show five different patterns (P1-P5) in five different samples. (**D**) PCR-SSCP bands of *GHR.E8* show three different patterns (P1-P3) in four different samples. (**E**) Sequences chromatogram spanning the site of c.380G>A (p.Arg(R)127Lys (K)) and c.387C>T (p.Gly(G)129) in E5. (**F**) Sequences chromatogram spanning the site of c.435A>G (p.Pro(P)145) in E6. (**G**) Sequences chromatogram spanning the site of c.836T>A (p.Phe(F)279Tyr(Y)) in E8. (**H**) Location of the predicted c.380G>A (p.Arg127Lys), c.387C>T (p.Gly129), c.435A>G (p.Pro145), and c.836T>A (p.Phe279Tyr) SNPs in the extracellular and transmembrane domains of the GHR protein. Blue cylinders indicate the exons (1–10).

In contrast, the other three loci (*GHR.E5*, *GHR.E6*, and *GHR.E8*) showed 5, 3, and 3 different genotypes, respectively (Figure 1A–D). Analysis of *GHR.E5* sequences showed 2 SNPs: A missense mutation c.380G>A that changed arginine to lysine amino acid (p.Arg127Lys) and was located in GHR extracellular domain (ECD), and another silent mutation c.387C>T (p.Gly129) (Figure 1E,H). One more silent mutation c.435A>G (p.Pro145) was detected in E6 (Figure 1F). E8 showed one missense mutation c.836T>A (p.Phe279Tyr) in GHR ECD which replaced phenylalanine (neutral aa) by tyrosine (polar un-charged aa) (Figure 1G,H). The locations of these SNPs were determined based on the published buffalo sequences (accession number NM_001290971). The sequence for the first three loci was assembled in one combined sequence and was submitted to GenBank (accession number KC107765), that of *GHR.E8* (less than 200 bp) is presented in Figure S3.

We then compared nucleotide sequences of *GHR.E5*, *GHR.E6*, and *GHR.E8* in Egyptian and Indian water buffaloes and other closely-related ruminant species (cattle, sheep, and goat) and we found that c.380G>A in E5 and c.387C>T in E6 were novel SNPs as they were present only in Egyptian buffaloes (Table S2). All other animals contained only the G alleles which subsequently were the ancestral (wild) alleles. Unlike Indian water buffaloes, *GHR.E5* locus in Egyptian buffaloes lacked c.348T>C and c.381A>C SNPs [12]. The only shared SNP between Egyptian and Indian buffaloes was the c.387C>T SNP. None of these previously described 5 SNPs were found in other ruminant species, so far. The c.836T>A in E8 was the only shared SNP between buffalo and cattle. At the protein level, the wild c.380G (Arg) allele was conserved in all species studied thus far, while the mutant c.380A(Lys) allele was only detected in Egyptian buffaloes (Table S2). This high degree of Arg conservation indicates that replacement of this aa by Lys may affect GHR function.

### 3.2. Analysis of Genotype and Haplotype Frequencies

The genotype and allele frequencies are shown in Table 2. Wild alleles frequencies (c.380G:0.745, c.387C:0.53, c.435A:0.555, and c.836T:0.815) and their homozygous genotypes (GG: 0.585, CC: 0.32, AA: 0.35, and TT: 0.665, respectively) were higher than the mutant alleles (c.380A:0.255, c.387T:0.47, c.435G:0.445, and c.836A:0.185) and their corresponding homozygous genotypes (AA:0.095, TT:0.26, GG:0.24, and AA:0.035, respectively). The heterozygous genotypes showed lower frequencies (c.380GA: 0.32 and c.836TA: 0.30) than the wild homozygous genotypes. Low frequency for the mutant c.836A allele was also observed in cattle *GHR* [5,6]. However, only c.836TT and c.836TA genotypes were detected in Indian buffaloes [13], suggesting that Indian buffaloes only fixed the T allele. The absence of c.836AA genotype in Indian buffaloes could be attributed to a few numbers of genotyped buffaloes (136 animals).

The different genotypes of the c.380G>A, c.387C>T, c.435A>G SNPs deviated from Hardy–Weinberg Equilibrium (HWE) (*p* < 0.05), while those of the c.836T>A SNP fit HWE (*p* > 0.05) (Table 2). Agreement with HWE indicates the absence of either natural or artificial selection for c.836T>A SNP in Egyptian buffaloes. Both c.380G>A and c.836T>A SNPs exhibited moderate genetic diversity with effective allele numbers (Ne) values of 1.61 and 1.43, polymorphism information content (PIC) values of 0.31 and 0.26, and heterozygosity (He) values of 0.38 and 0.30, respectively (Table 2). This moderate diversity makes these 2 SNPs more valuable for MAS. Moreover, these 2 SNPs also showed a very strong linkage (D’ = 1; r2 = 0.078), as revealed by data obtained from pair-wise linkage disequilibrium (LD) analysis (Figure S4). This suggests co-inheritance of c.380G>A and c.836T>A SNPs and their dependent effect on milk performance. For this reason, we further studied the frequencies of their four haplotypes (GT, GA, AT, and AA) and found higher frequencies in animals carrying the two wild alleles (GT: 0.64) and lower frequencies in the other haplotypes (GA:0.12, AT: 0.17, and AA: 0.07). This indicates a lower distribution of mutant alleles among the 400 Egyptian buffaloes included in our study.

### 3.3. Association of Genotypes and Haplotypes with Milk Yield and Composition

Although the two silent mutations produced by c.387C>T and c.435A>G SNPs could affect milk production traits, they were excluded from association study because they did not fit HWE and were not in LD with each other or with other studied SNPs (Figure S4). For these 2 SNPs, we have checked the ratio of observed to expected number of heterozygote carriers, which were 0.84 and 0.82, respectively. This indicated that the departure from HWE was mainly associated with loss of heterozygotes which is less likely to occur in case of genotyping errors [27]. From the data we have considering population substructure, there was no clear evidence of population stratification. So, these deviations were more likely to be associated with attempts of purifying selection bearing in mind that our study population was not practicing inbreeding techniques. 

Herein, we studied the association of the two missense SNPs (c.380G>A and c.836T>A), individually and in combination, with milk production traits. The mutant c.380A allele, and c.380AA genotype were significantly associated with higher milk yield, fat %, protein %, and 305 day-milk, fat and protein yield as compared to the wild c.380G allele and c.380GG genotype (Table 3). The heterozygous animals (c.380GA) had non-significantly higher fat % protein %, and 305 day-milk, fat and protein yield than c.380GG-genotype animals. However, no significant association was noticed between any of these genotypes and lactose % and total solid %.

Animals with the mutant c.836A allele, and c.836AA genotypes had significantly higher milk yield but lower fat %, protein %, and 305 day-milk, fat and protein yield relative to animals with the wild c.836T allele and c.836TT genotypes (Table 4). Similar associations with milk yield and milk composition for this SNP were also reported in cattle where cows with A allele produced higher milk yield, but lower fat and protein yields than those with T allele [5,6,7,10]. It is likely that high frequencies of T allele in buffaloes might be associated with the higher milk fat content and low milk yield in buffalo as compared to cattle [13]. The lower frequencies of the A allele could be attributed to its association with another unfavorable trait such as longer calving interval [9], which is probably selected against to a greater extent than for superior milk production. Recently, c.836T>A SNP was also suggested to be a functional SNP for lactation persistency, animal ability to maintain superior milk yield after milk peak [28]. This indicates a potential superior impact for this SNP on milk production in cattle. Additionally, cows with A allele had reduced somatic cell score compared to those with the T allele, suggesting the importance of selecting cows with A allele to enhance resistance to mastitis [6,9]. In general, high genetic progress for milk yield (which is the main goal for breeders) could be obtained from MAS when animals with the rare allele (A in this case) were selected. However, due to the high frequency of the T allele, this SNP could not have a significant effect on MAS for milk fat %, protein %, and 305 day-milk, fat and protein yield [29]. Consequently, c.836T>A SNP alone could not be the responsible mutation for milk production QTL, and this SNP is likely in linkage disequilibrium with other SNPs in the same gene or other related genes.

This prompted us to study the association of c.380G>A and c.836T>A four haplotypes (GT, GA, AT, and AA) with milk performance (Table 5). Animals with mutant AA haplotypes produced significantly higher milk yield, fat %, protein %, and 305 day-milk, fat and protein yield than animals with wild GT haplotypes. Animals with heterozygous haplotypes (AT and GA) also produced significantly higher milk yield, fat %, protein %, and 305 day-milk, fat and protein yield than those with wild haplotypes, but at levels lower than animals with mutant alleles. Overall, the mutant haplotype was the most favorable one for higher milk performance (high milk yield, fat %, protein %, and 305 day-milk, fat and protein yield). These findings also confirm the importance of studying the inheritance of haplotypes rather than individual SNPs [30].

### 3.4. Association of GHR Polymorphisms with Gene and Protein Expression

**Figure 2:**
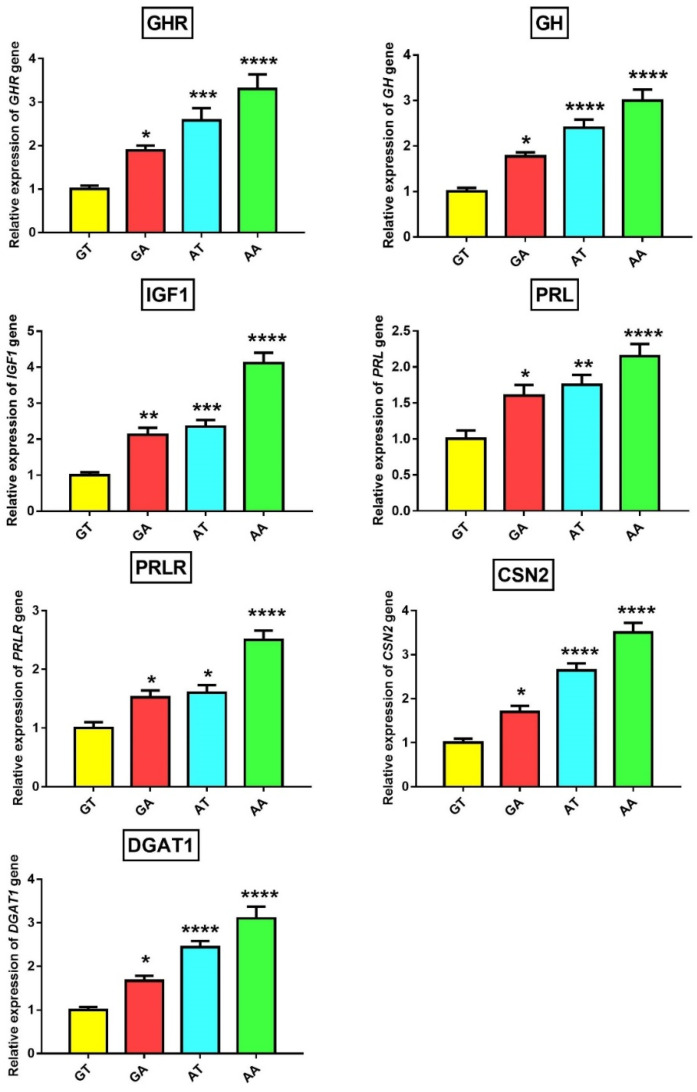
Associations between GT, GA, GT, and AT haplotypes and expression of *GHR*, *GH*, *IGF1*, *PRL*, *PRLR*, *CSN2*, and *DGAT1* genes in milk somatic cells as detected by qRT-PCR. The *β-actin* gene was used as an internal control and data are presented as mean of gene expression fold change ± SEM (n = 9/haplotype) with wild haplotype GT considered as the baseline. * *p* < 0.05, ** *p* < 0.01, *** *p* < 0.001, and **** *p* < 0.0001. All *p*-values were obtained using Bonferroni’s correction for comparison of all haplotypes against wild haplotype GT.

**Figure 3:**
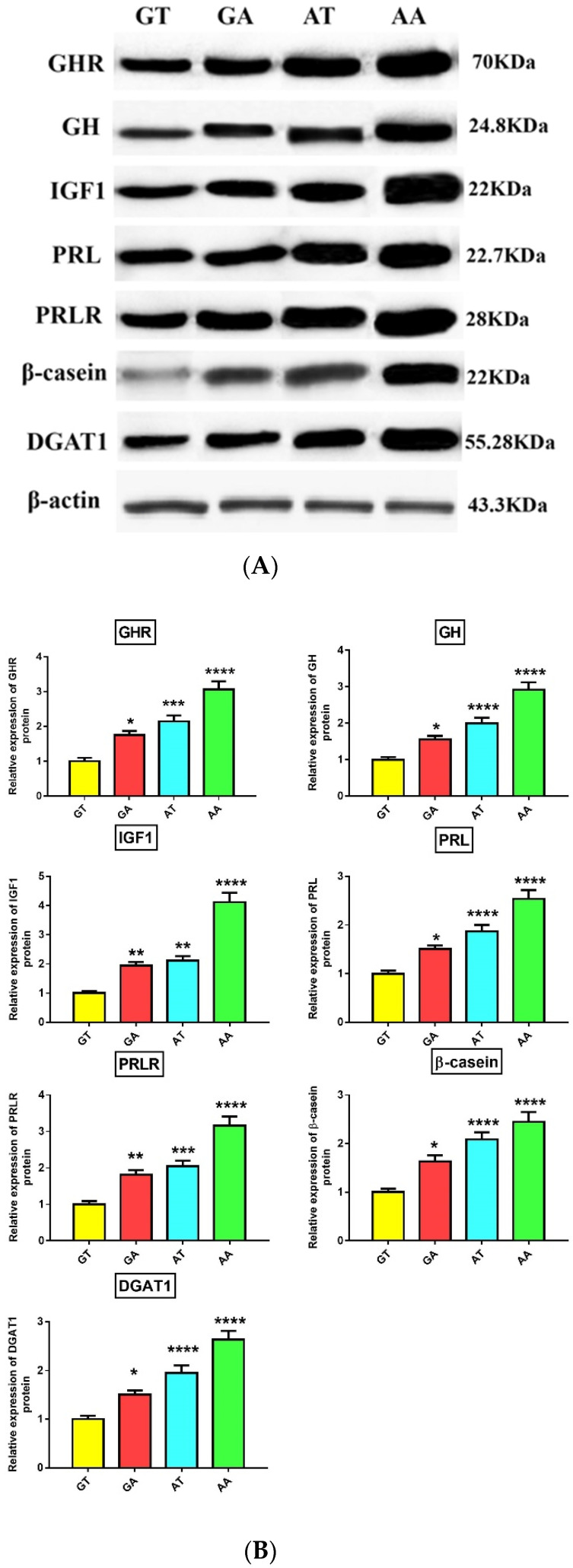
(**A**) Associations between GT, GA, AT, and AA haplotypes and expression of GHR, GH, IGF1, PRL, PRLR, β-casein, and DGAT1 proteins in the milk somatic cells as detected by western blot. The β-actin protein was used as an internal control. (**B**) Band quantification of proteins displayed in (A). Data are presented as mean of protein expression fold change ± SEM (n = 9/haplotype) with GT haplotype considered as the baseline. * *p* < 0.05, ** *p* < 0.01, *** *p* < 0.001, and **** *p* < 0.0001. All *p*-values were obtained using Bonferroni’s correction for comparison of all haplotypes against wild haplotype GT.

The missense mutations in a gene can affect phenotypic traits through alteration of gene and protein expression [31]. Subsequently, we checked associations between the four haplotypes and the expression of genes- and proteins-related to milk yield (GHR, GH, IGF1, PRL, PRLR), milk protein (CSN2/β-casein), and milk fat (DGAT1) in milk somatic cells (SCs). Notably, we found significantly (*p* < 0.05) higher expression levels of these genes and proteins in animals with mutant AA haplotypes than other haplotypes (Figure 2 and Figure 3). Also, animals carrying heterozygous haplotypes (GA and AT) had significantly higher expression levels of these genes and proteins than wild GT haplotype animals. These results were consistent with association study and both denoted higher milk yield, fat %, protein %, and 305 day-milk, fat and protein yield in the mutant AA-haplotype animals. In general, animals carrying AA haplotypes showed superior milk performance accompanied by greater expression of GHR, GH, IGF1, PRL, PRLR, CSN2/β-casein, and DGAT1 genes and proteins in SCs.

GH has a potential galactopoietic effect in ruminants and together with other members of the somatotrophic axis, such as IGF1, plays a vital role in the initiation and maintenance of milk yield, animal growth, and fertility [32]. Most, or even all, of these effects are mediated through binding with GH binding site in GHR ECD with subsequent activation of JAK2/STAT signaling pathway and their downstream targets such as IGF1 [33]. So, any change in the GH binding region of GHR would affect the GH-GHR- JAK2/STAT signal pathway and activity of GH on the target cell [34]. The c.380G>A SNP is located in GHR ECD (Figure 1H) and therefore G(Arg) to A(Lys) substitution could negatively affect the binding with GH and GH-GHR-JAK2/STAT signaling pathway. Even though c.836T>A SNP is located in the transmembrane domain slightly away from GH binding site, T(Phe) to A(Tyr) substitution could change the functional structure of the transmembrane domain which could further affect the adjacent extracellular and intracellular domains. In support, the same SNP in cattle resulted in significant differences in the GHR binding capacity and subsequently influenced the physiological properties of the receptor [35]. The galactopoietic influence of GH in dairy animals is mediated mainly by IGF1 [36]. Notably, GH and IGF1 were significantly increased in SCs in mutant AA-haplotype buffaloes. Therefore, c.380G>A and c.836T>A SNPs may directly or indirectly (through linkage with other causative SNPs) change GH which could further induce IGF1 expression, leading to higher milk production in these buffaloes. To the best of our knowledge, this study is the first to report an association between these 2 SNPs and expression of GHR, GH, and IGF1 genes and proteins in buffalo milk SCs.

Although most previous studies concluded that c.836T>A could be a causative SNP for the observed milk production and composition traits, we could not exclude the effects of other linked SNPs in the *GHR* (such as c.380G>A) or other genes in regions close to c.836T>A locus. A good example of these close genes is *PRLR*, which locates approximately 7 Mb from the *GHR* and its SNPs associated with milk yield, and fat and protein content in cattle [8,37]. Both GH and PRL hormones play a crucial role in the initiation and maintenance of lactation in addition to the development of the mammary gland and the development of their genes from a common ancestral gene [33,38]. Although GH and PRL hormones have different functions, their functions strongly overlap during lactation [33,38]. Similarly, mutations of *PRLR* and *GHR* could mutually participate in the same phenotypic effect. Therefore, the effect of c.380G>A and c.836T>A SNPs of *GHR* on *PRL* and *PRLR* could be indirect through linkage with other SNPs in the *PRLR* and/or *PRL* gene. Similarly, SNPs-induced reduction of *PRLR* expression could disrupt the PRL/PRLR-JAK2/STAT signaling pathway and affect their downstream targets such as *CSN2* [39]. This also may explain change of CSN2/β-casein expression in SCs due to c.380G>A and c.836T>A SNPs of *GHR*. To the best of our knowledge, this study is the first to report an association between these 2 SNPs and expression of PRL, PRLR, and CSN2/β-casein genes and proteins in buffalo milk SCs.

DGAT1 plays an important role in the formation of milk fat [40]. To date, there have been no studies that have investigated whether DGAT1 is a downstream target for the JAK2/STAT signaling pathway during lactogenesis and adipogenesis. Thus, the effect of c.380G>A and c.836T>A SNPs on DGAT1 expression could be indirect. To our knowledge, this study may be the first to report the association between these 2 SNPs and expression of the DGAT1 gene and protein in buffalo milk SCs. Previous studies have reported a significant association between the c.836T allele and the increased levels of C15:0, C17:0, and C18:1 trans10 fatty acids in cattle milk fat [41]. Similarly, Li et al. [42] found a significant association between a silent mutation (at a chromosomal number of 32030332) in intron 2 of the *GHR* gene and C18:0 concentration in cattle milk. As milk C15:0, C17:0, and C18:1 trans10 fatty acids are synthesized by ruminal microbes [43], the association with these SNPs could be indirect. C18:1 trans10 fatty acids may be involved in pathways regulating the expression of lipogenesis-related genes [44]. These genetic markers could allow breeders to select animals that produce high milk yield with a healthier fatty acid content. Further investigations are required to check whether c.380G>A and c.836T>A SNPs of *GHR* would affect the fatty acids composition of buffalo milk.

In the present study, 2 SNPs of *GHR* showed a significant association with milk performance in Egyptian water buffaloes. However, we could not neglect the effect of other SNPs in other regions of this gene. Indeed, some other SNPs in the promoter of bovine *GHR*, such as rs132896414, ss159831013 had a significant effect on milk protein % and milk yield, respectively [5,9,45]. Therefore, further investigations to screen the whole sequence of the *GHR* gene (including coding and non-coding sequences) for putative polymorphisms in independent large populations along with functional genomic analyses are required.

## 4. Conclusions

To the best of our knowledge, this is the first study to report the presence of a novel non-synonymous c.380G>A in the extracellular domain of GHR and to confirm the presence of the well-known cattle SNP c.836T>A in Egyptian buffalo. These two SNPs were associated with milk yield, fat %, protein %, and 305 day-milk, fat and protein yield, with higher performance for mutant AA-haplotype animals. This superior effect was accompanied by higher expression of GHR, GH, IGF1, PRL, PRLR, CSN2/β-casein, and DGAT1 genes and proteins in milk somatic cells. Thus, selection for AA haplotypes could improve milk production traits in Egyptian buffaloes.

## Figures and Tables

**Table 1:** Primers used in conventional and qRT-PCR.

Gene	Forward Primer	Reverse Primer	Ta (°C)	Localization *	Size (bp)	Experiment
GHR.E4	AGGACCATCCATTACCCTCCTGATTT	TCCATTCCCATCACTGCATGAC	56	Exon4 (E4), introns(I) 2, 3	265	SNPs detection
GHR.E5	AGGAGCTGGCACCTTATATGCAGT	CCCCGCTTATGTAATCTAAAGCCATGT		E5, I4, I5	472	
GHR.E6	ACTGATTCTCTGCTGAAATGCACAGT	CCATTTTCCACTGGGTCTCATTCAGT		E6, I5	205	
GHR.E8	CTTTGGAATACTTGGGCTAG	CACTTCACTCAGGATTCAC		E8, I8	166	
GHR	CCAGCTTTCCTTGTCAGAGCA	TGTGATTAGCCCCATCTGTCC	60	E2 and E3	148	Relative expression by qRT-PCR
PRLR	AACCATTGAGACTGGCAGGG	AAGGGGGTTTTGTCTTGGGG		E10	114	
PRL	GCATGCTTGGCTCTAATGGG	TGTCAGTTTCTGCTATTTGTGAC		Coding sequences	186	
GH	CAGCCATCTGTTGTTTGCCC	CCCCCAGAATAGAATGACACC			130	
IGF1	TTGGTGGATGCTCTCCAGTTC	AGCAGCACTCATCCACGATTC			218	
β-casein	AATCTGCACCTTCCTCTGCC	ACTGAGAAAGGGACAGCACG			109	
DGAT1	GGTCCGGGACACAGACAAG	CTGCTGAAGCCACTGTCAGA			111	
β-actin	CGACAACGGCTCCGGCATGT	CTCCTCAGGGGCCACACGGA			211	

* PRLR loci were determined based on published buffalo sequences (KC107765.1, EF207441 and NM_001290971.1). qRT-PCR, quantitative reverse transcription polymerase chain reaction; SNPs, single nucleotide polymorphisms; Ta, annealing temperature.

**Table 2:** Genotypic and allelic frequencies, value of *χ^2^* test, and diversity parameter of c.380G>A, c.387C>T, c.435A>G, and c.836T>A SNPs of buffalo *GHR*.

SNP	Genotype Frequency (Number)			Allele Frequency		χ^2^ (*p*-Value)	He	Ne	MAF	PIC
c.380G>A	GG	GA	AA	G	A	5.16 (0.023)	0.38	1.61	0.255	0.31
	0.585 (234)	0.32 (128)	0.095 (38)	0.745	0.255					
c.387C>T	CC	CT	TT	C	A	5.09 (0.024)	0.49	1.99	0.47	0.37
	0.32 (128)	0.42 (168)	0.26 (104)	0.53	0.47					
c.435A>G	AA	AG	GG	A	G	6.75 (0.009)	0.49	1.97	0.445	0.37
	0.35 (140)	0.41 (164)	0.24 (96)	0.555	0.445					
c.836T>A	TT	TA	AA	T	A	0.012 (0.914)	0.30	1.43	0.185	0.26
	0.665 (266)	0.30 (120)	0.035 (14)	0.815	0.185					

D’, linkage disequilibrium coefficient; He, gene heterozygosity; HWE, Hardy–Weinberg equilibrium; MAF, minor allelic frequency; Ne, effective allele numbers; PIC, polymorphism information content; χ^2^, Chi-square value.

**Table 3:** Association between individual c.380G>A SNP and milk yield and quality traits.

Traits	GG (n = 234)	GA (n = 128)	AA (n = 38)
Milk yield (kg, 305 day)	2048.33 ± 46.28 ^b^	2052.04 ± 49.50 ^b^	2330.48 ± 48.27 ^a^
Fat percentage	6.12 ± 0.12 ^Bb^	6.45 ± 0.13 ^Bb^	7.26 ± 0.14 ^Aa^
305 day-fat yield (kg)	130.35 ± 2.74 ^Bb^	132.41 ± 2.20 ^Bb^	151.19 ± 5.07 ^Aa^
Protein percentage	4.05 ± 0.10 ^Bb^	4.32 ± 0.09 ^Bb^	4.81 ± 0.12 ^Aa^
305 day-protein yield (kg)	84.36 ± 2.19 ^Bb^	86.65 ± 2.34 ^Bb^	104.48 ± 4.60 ^Aa^
Lactose percentage	5.32 ± 0.17	5.14 ± 0.25	5.08 ± 0.19
Total solid percentage	16.42 ± 0.38	16.60 ± 0.42	17.05 ± 0.45

Data are expressed as least squares means ± SEM. Different lowercase letters indicate significant differences between genotypes (*p* < 0.05). Different uppercase letters indicate significant differences between genotypes (*p* < 0.01).

**Table 4:** Association of individual c.836T>A SNP with milk yield and quality traits.

Traits	TT (n = 266)	TA (n = 120)	AA (n = 14)
Milk yield (kg, 305 day)	2032.33 ± 30.25 ^b^	2058.80 ± 33.22 ^b^	2369.25 ± 31.47 ^a^
Fat percentage	7.09 ± 0.10 ^Aa^	6.55 ± 0.09 ^Ab^	6.00 ± 0.08 ^Bc^
305 day-fat yield (kg)	144.17 ± 0.68 ^Aa^	135.63 ± 0.59 ^Bb^	136.80 ± 0.62 ^b^
Protein percentage	4.92 ± 0.08 ^Aa^	4.39 ± 0.07 ^Ab^	4.15 ± 0.06 ^Bb^
305 day-protein yield (kg)	102.67 ± 0.74 ^Aa^	90.83 ± 0.62 ^Bb^	95.01 ± 0.60 ^b^
Lactose percentage	5.49 ± 0.28	5.30 ± 0.21	5.11 ± 0.10
Total solid percentage	17.26 ± 0.34	17.10 ± 0.31	16.87 ± 0.49

Data are expressed as least squares means ± SEM. Different lowercase letters indicate significant differences between genotypes (*p* < 0.05). Different uppercase letters indicate significant differences between genotypes (*p* < 0.01).

**Table 5:** Association between different haplotypes (GT, GA, AT, and AA) of *GHR* and milk yield and quality traits.

Traits	GT (n = 256)	GA (n = 48)	AT (n = 68)	AA (n = 28)
Milk yield (kg, 305 day)	2090.48 ± 43.82 ^b^	2340.17 ± 40.80 ^a^	2315.48 ± 44.19 ^a^	2438.37 ± 43.05 ^a^
Fat percentage	6.03 ± 0.12 ^Bb^	6.78 ± 0.10 ^a^	7.04 ± 0.16 ^Aa^	7.38 ± 0.15 ^Aa^
305 day-fat yield (kg)	126.16 ± 1.94 ^Cc^	158.35 ± 2.20 ^Bb^	163.20 ± 2.16 ^Bb^	179.38 ± 2.54 ^Aa^
Protein percentage	4.05 ± 0.08 ^Bb^	4.51 ± 0.07 ^a^	4.62 ± 0.09 ^Aa^	4.98 ± 0.11 ^Aa^
305 day-protein yield (kg)	84.66 ± 1.04 ^Bc^	105.75 ± 1.13 ^Ab^	106.47 ± 1.15 ^Ab^	114.56 ± 1.37 ^Aa^
Lactose percentage	5.10 ± 0.14	5.30 ± 0.16	5.24 ± 0.23	5.51 ± 0.34
Total solid percentage	17.24 ± 0.39	16.85 ± 0.30	17.00 ± 0.34	17.37 ± 0.39

Data are expressed as least squares means ± SEM. Different lowercase letters indicate significant differences between genotypes (*p* < 0.05). Different uppercase letters indicate significant differences between genotypes (*p* < 0.01). All *p*-values were obtained using Bonferroni’s correction for comparison of all haplotypes against wild haplotype GT.

## Supplementary Materials

The following are available online at https://www.mdpi.com/2076-2615/10/7/1203/s1, Figure S1: A representative ethidium bromide stained agarose gel of PCR products representing amplification of *GHR.E4* with a size of 265 bp in 4 different Egyptian buffaloes. M represents 100 bp DNA ladder, Figure S2: PCR-SSCP bands of the *GHR.E4* in 3 buffaloes show similar pattern, Figure S3: A representative sequence chromatogram of *GHR.E4* shows its structure: Exon 4 (yellow bar), part of introns 3 and 4 (cyan bars), and forward and reverse primers (green arrows), Figure S4: Pair-wise linkage disequilibrium analysis revealed a very strong linkage (D’= 1 (100%) and r2 = 0.078, as indicated by strong red diamond)) between c.380G>A and c.836T>A SNPs. The percentages of linkage between the four SNPs were presented within associated diamonds and the intensity of red color indicates the strength of linkage. D’, linkage disequilibrium coefficient, Table S1: Dilutions and sources of antibodies used in western blot, Table S2: Comparative analysis of SNPs detected in *GHR.E5*, *GHR.E6*, and *GHR.E8* loci between Egyptian water buffalo (this study) and Indian buffaloes as well as the closely related ruminant species.
