# Supplementary material for: Association of GHR Polymorphisms with Milk Production in Buffaloes
Source: Animals (Basel). 2020 Jul 15;10(7):1203. doi: 10.3390/ani10071203 (PMC7401641; doi:10.3390/ani10071203)
Supplement: Supplementary file 1 [file animals-10-01203-s001.pdf]

animals

MDPI

Supplementary data

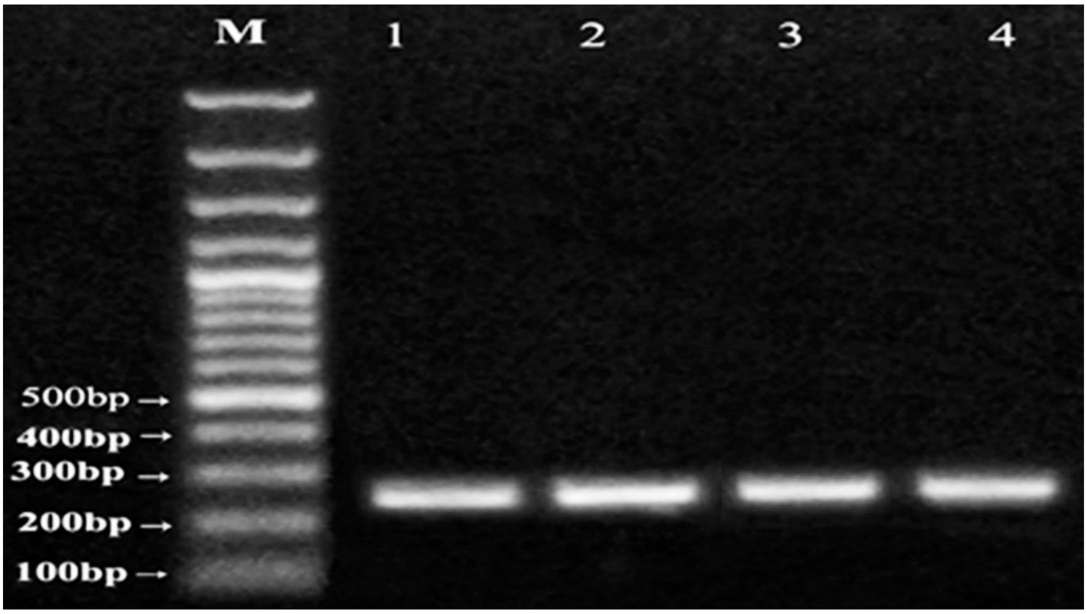

Figure S1. A representative ethidium bromide stained agarose gel of PCR products representing amplification of *GHR.E4* with a size of 265 bp in 4 different Egyptian buffaloes**.** M represents 100 bp DNA ladder.

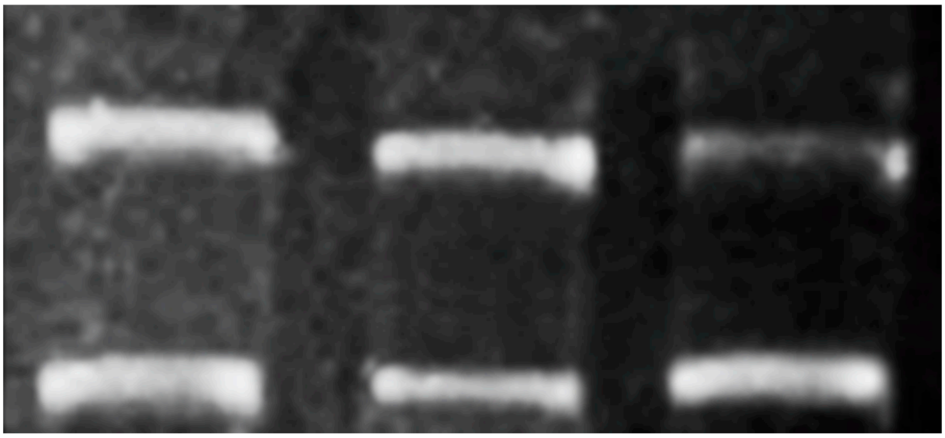

Figure S2. PCR-SSCP bands of the *GHR.E4* in 3 buffaloes show similar pattern.

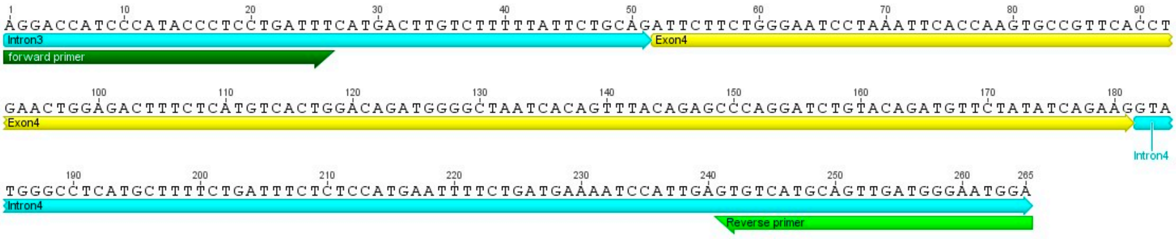

Figure S3. A representative sequence chromatogram of *GHR.E4* shows its structure: exon 4 (yellow bar), part of introns 3 and 4 (cyan bars), and forward and reverse primers (green arrows).

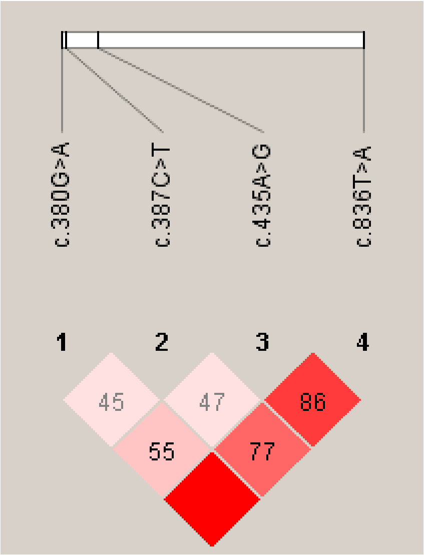

Figure S4. Pair-wise linkage disequilibrium analysis revealed a very strong linkage [D'= 1 (100%) and r2 = 0.078, as indicated by strong red diamond)] between c.380G>A and c.836T>A SNPs. The percentages of linkage between the four SNPs were presented within associated diamonds and the intensity of red color indicates the strength of linkage. D', linkage disequilibrium coefficient.

Table S1. Dilutions and sources of antibodies used in western blot.

	**Name**	**Dilution**	**Size (KDa)**	**Source**	**Catalogue number**
1	Anti-IGF1 primary antibody (ab)	1:300	22	Novus Biologicals Europe, UK	NBP2-16929
2	Bovine anti-GH1 polyclonal primary ab	1:200	24.8	MyBioSource, San Diego, USA	MBS627535
3	Anti-GHR polyclonal primary ab	1:250	70	MyBioSource, San Diego, USA	MBS9608786
4	Anti-β-actin primary ab	1:200	43.3	Santa Cruz Biotechnology, Inc., USA	sc-47778
5	Bovine anti-PRL polyclonal primary ab	1:300	22.7	Cloud-Clone Corp., USA	PAA846Bo01
6	Bovine anti-PRLR primary ab	1:300	28	MyBioSource, San Diego, USA	MBS2026486
7	Bovine anti-DGAT1 polyclonal primary ab	1:250	55.28	MyBioSource, San Diego, USA	MBS243054
8	Sheep anti-Bovine casein polyclonal primary ab	1:1000	22	MyBioSource, San Diego, USA	MBS560643
9	Horseradish peroxidase conjugated anti-rabbit IgG secondary ab	1:5000		Santa Cruz Biotechnology, Inc., USA	sc-2030

Table S2. Comparative analysis of SNPs detected in *GHR.E5, GHR.E6*, and *GHR.E8* loci between Egyptian water buffalo (this study) and Indian buffaloes as well as the closely related ruminant species.

Locus	Exon 5				Exon 6	Exon 8
Nucleotide position*	348	380	381	387	435	836
SNP	T>C	G>A	A>C	C>T	A>G	T>A
Amino acid position	116	127	127	129	145	279
Amino acid change	Ser	Arg/Lys	Arg/Ser	Gly	Pro	Phe/Tyr
Codon change	TCT TC**C**	AGA A**A**A	AGA AG**C**	GGC GG**T**	CCA CC**G**	TTT T**A**T
Egyptian Bubalus bubalis[1]	TCT	AGA A**A**A	AGA	GGC GG**T**	CCA CC**G**	TTT T**A**T
Indian Bubalus bubalis[2]	TCT TC**C**	AGA	AGA AG**C**	GGC GG**T**	CCA	TTT T**A**T
Indian Bubalus bubalis[3]	TCT	AGA	AGA	GGC	CCA	TTT T**A**T
Bos taurus[4]	TCT	AGA	AG**C**	GG**C**	CCA	TTT T**A**T
Capra hircus[5]	TC**C**	AGA	AG**C**	GG**T**	CCA	TTT
Ovis aries[6]	TC**C**	AGA	AG**C**	GG**T**	CCA	TTT

* According to coding sequences of NM_001290971.1. The blue nucleotides indicate the mutant alleles.

[1] the present study; [2] NW_005785241.1 (Sur), JN882283.1, JN257666.1; [3] NM_001290971.1, EF207441; [4]AY748827.1; [5]AH015394.2; [6]NM_001009323.2.
